# Hyperelastic Material Parameter Determination and Numerical Study of TPU and PDMS Dampers

**DOI:** 10.3390/ma14247639

**Published:** 2021-12-11

**Authors:** Carina Emminger, Umut D. Çakmak, Rene Preuer, Ingrid Graz, Zoltán Major

**Affiliations:** 1 Christian Doppler Laboratory for Soft Structures for Vibration Isolation and Impact Protection (ADAPT), Institute of Polymer Product Engineering, Johannes Kepler University Linz, Altenbergerstrasse 69, 4040 Linz, Austria; zoltan.major@jku.at; 2 Christian Doppler Laboratory for Soft Structures for Vibration Isolation and Impact Protection (ADAPT), School of Education, STEM Education, Johannes Kepler University Linz, Altenbergerstrasse 69, 4040 Linz, Austria; rene.preuer@jku.at (R.P.); ingrid.graz@jku.at (I.G.)

**Keywords:** hyperelastic material modelling, material parameter determination, TPU, PDMS, damper structures

## Abstract

Dampers provide safety by controlling unwanted motion that is caused due to the conversion of mechanical work into another form of energy (e.g., heat). State-of-the-art materials are elastomers and include thermoplastic elastomers. For the polymer-appropriate replacement of multi-component shock absorbers comprising mounts, rods, hydraulic fluids, pneumatic devices, or electro-magnetic devices, among others, in-depth insights into the mechanical characteristics of damper materials are required. The ultimate objective is to reduce complexity by utilizing inherent material damping rather than structural (multi-component) damping properties. The objective of this work was to compare the damping behavior of different elastomeric materials including thermoplastic poly(urethane) (TPU) and silicone rubber blends (mixtures of different poly(dimethylsiloxane) (PDMS)). Therefore, the materials were hyper- and viscoelastic characterized, a finite element calculation of a ball drop test was performed, and for validation, the rebound resilience was measured experimentally. The results revealed that the material parameter determination methodology is reliable, and the data that were applied for simulation led to realistic predictions. Interestingly, the rebound resilience of the mixture of soft and hard PDMS (50:50) wt% was the highest, and the lowest values were measured for TPU.

## 1. Introduction

Damping systems are indispensable in daily life and are applied in products that are used daily, such as in cars to make driving more comfortable, in machines to reduce noise, in sports equipment and footwear (e.g., the soles of shoes to protect human joints) [1], among other applications. The (motion) damping mechanism can be separated into active and passive as well as into semi-active (a combination of both) [2,3]. Active damping is required because passive damping causes an increase in a system’s transmissibility at high frequencies [3]. Furthermore, active damping enables the nearly perfect damping of a system over the entire application range, which is the case in sport cars [4]. In general, multi-material compositions with properties that are able to be adjusted by physical fields (e.g., magnetic, electric, thermal, etc.) are used in active damping. The most common materials are magnetically polarizable particle-filled elastomers with the ability to change their behavior rapidly, continuously, and reversibly using an applied magnetic field [2,5]. The adjustability of an elastomer’s mechanical behavior within magnetic fields is measured by the ratio of the magneto-induced shear modulus and the zero-field modulus (ΔG/G0) and is characterized by the dimensionless parameter representing the relative magnetorheological (MR) effect [2]. The MR effect exhibits the (same) inherent viscoelastic behavior as the matrix material (elastomer) and is therefore a function of the loading rate (time) and temperature. In addition, the effect is more pronounced when the elastomer is softer (even higher for magnetorheological fluids). It can be shown that the softer (compliant mechanical behavior) the matrix is, the higher the MR effect is, a response that is due to the fact that the particles that are within the matrix elastomer have more unrestricted movement [2,6]. However, the material data for the physical field-dependent material properties are not easily available; consequently, the interplay of the inherent viscoelasticity, the particles, and the physical field represents a vivid field of research that is currently lacking insight. In contrast, passive damping represents the response of a material due to a disturbance in the system [2]. The damping properties depend on the composition of the material and, in the case of elastomers, the material behavior is inherently viscoelastic, with a pronounced temperature and frequency dependency. However, they are usually not adjustable when steady-state operation is taking place under isothermal conditions [2,7]. As such, an understanding of the fundamental viscoelastic behavior of elastomers is of particular importance in order to understand the dissipation capability upon external loading. A convenient method that can be used to characterize this behavior is the dynamic mechanical analysis within the experimental window in which temperatures, frequencies, and strain amplitudes are analyzed [3,7,8]. The complex dynamic modulus of different elastomers can be split into a real and an imaginary part (storage Young’s modulus E′ and loss Young’s modulus E″). E″ as well as the loss factor (tanδ = E″/E′) are directly related to the damping capacity (ratio of dissipation energy and elastic energy, U_d_/U_e_); the higher E″ (tanδ) is, the higher the damping affect is [9,10,11]. For the elastomer-appropriate design of damping systems, polymer product engineering methodologies are needed. Otherwise, the utilization of both active and passive dampers in engineering applications is limited to non-structural parts. To overcome this limitation, this work applies systematic experimental and material parameter determination procedures that are based on the methods published in [7,12,13].

The main objective of this study was to analyze the damping behavior of silicone elastomers (PDMS) and thermoplastic poly(urethanes) (TPU). These materials are candidate materials for damping applications, and various research studies have highlighted their damping performance (e.g., [14,15]). The formulations of these materials are tailorable (see [14,16]) in order to accommodate the required stiffness to maintain structural stability under their own weight, while under dynamic excitation, the unwanted vibrations are attenuated.

A methodology is presented that includes hyper- as well as viscoelastic characterization and material parameter determination for finite element analyses (FEA). Moreover, the rebound resilience of various elastomer dampers was determined experimentally. The finite element (FE) calculations of a ball-drop test were performed with material data that were obtained from hyper- as well as viscoelastic characterizations. Finally, to validate the FE model, rebound resilience experiments were conducted and were compared with the results from the simulation.

## 2. Materials and Methods

First, the hyperelastic and viscoelastic behaviors of the TPU and PDMS were examined and by applying appropriate data reduction methods, the parameters for constitutive models were determined. These parameters were implemented in the structural analyses that were performed using finite element methods (FEM). The specimens and damping elements made of PDMS were molded under laboratory conditions, and those that were made of TPU were provided from the material supplier.

### 2.1. Materials

Two types of elastomeric materials were investigated: (i) TPU and (ii) PDMS.A wide range of thermo-mechanical properties with different damping properties are covered when these materials are used (e.g., hardness ranges from Shore D50 down to Shore 00–30). These materials are ideal candidate materials for the desired function of a shock absorber rather than for structural damping. They reveal a stiffness that is suitable for the structure to be able to carry its own weight, provide high conformability under external loading, and sufficient damping capacity to fulfill the damping design objective. Furthermore, the examined materials are ideal damping materials and are materials of choice for damping elements. For instance, TPUs are utilized for shoe soles to enhance comfort [1], and silicone rubbers have been used in optical systems or for sensors [17].

Both TPUs were based on aromatic isocyanate and poly(carbonatpolyol) and were provided by the material supplier (DMH Dichtungs- und Maschinenhandel GmbH, Styria, Austria). They had a hardness of Shore D46 (TPU1-MDx) and D50 (TPU1). The TPU1-MDx was filled with 5 wt% iron (Fe) particles with a mean diameter of ⌀10 μm. Prior to the compounding of the Fe-particles into the TPU1 matrix, the surfaces of the particles were activated to improve the adhesion with the matrix. The purpose of the fillers is to enable detectability and electro-active as well as magneto-active behaviors, and even contactless communication and data transfer applications are feasible with these types of fillers. Furthermore, TPU1 filled with Fe particles is particularly interesting for active damping applications; however, without a magnetic field, the material behavior has to be characterized in order to gain insights into the effect that the Fe particles have on the macroscopic mechanical properties. In general, both TPUs have been developed for a service temperature range from −20 °C up to 115 °C, are FDA certified, and are resistant against hot water, oils, and ozone as well as synthetic and natural ester. They were provided as injection molded plates (90 mm × 40 mm) and cylinders (⌀45 mm × 150 mm). The temperature profile during the injection molding process was in the range of 190–235 °C, and the mold was cooled afterwards.

For the silicone rubber blends, a mixture of two different PDMS with Shore 00–30 Ecoflex^®^, from Smooth-on Inc., Macungie, PA, USA; referred to as Ecoflex in the following) and Shore A43 (Sylgard™ 184 from DowDuPont Inc., Wilmington, NC, USA; referred to as Sylgard in the following) were casted as sheets and cylinders. During the casting process, the PDMS formulations were degassed with a desiccator, and they were further cured at a temperature of 65 °C for 24 h. Previously, both of the liquid components that were used to produce the Sylgard (A and B) were mixed with a weight ratio of 10:1, and both components for the Ecoflex (A and B) were mixed 1:1 (wt%). Both materials have similar damping capacities (or loss factors tanδ) but different stiffnesses (Young’s moduli). However, they are miscible, resulting in blends with varying stiffnesses depending on the mixing ratio [18]. For this work, one blend in addition to the neat PDMS was characterized and analyzed. The PDMS formulations were named after the incorporated fraction of the Ecoflex, starting from neat Ecoflex (100E) with a blend of 50 wt% (50E) and neat Sylgard (0E).

### 2.2. Methods

**Figure 1:**
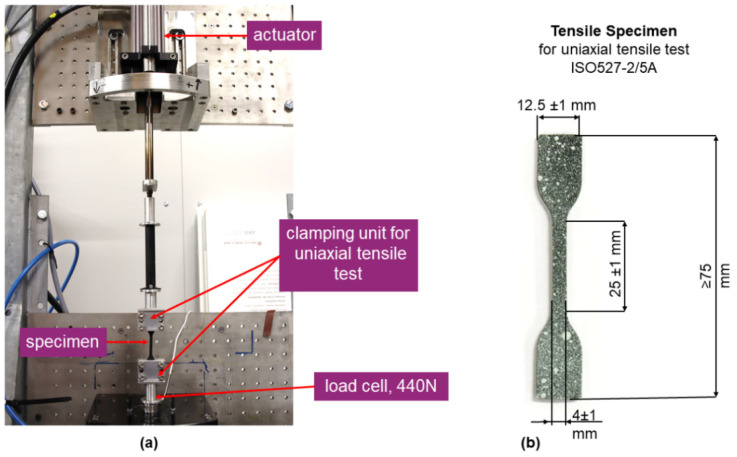
(**a**) Set-up of the uniaxial tensile test; (**b**) geometry of the used specimen according to DIN-EN-ISO-527-5A.

For the hyper- and viscoelastic characterization, the TPU specimens were stamped from molded plates, and the PDMS blends were casted under laboratory conditions. The uniaxial tension tests were performed with an electromechanical testing system (TestBench, Bose Corp., ElectroForce Systems Group, Eden Prairie, MN, USA) under isothermal conditions at room temperature and at three different loading rates (0.1 mm/s; 1 mm/s; 10 mm/s). The specimen geometry as well as the setup are shown in Figure 1. The measurements were displacement controlled, and the force was recorded with a 440 N load cell (WMC-100lbf, Interface Inc., Scottsdale, AZ, USA). The strains were derived by a LVDT (linear variable differential transformer, AD598, Analog Devices Inc., Wilmington, MA, USA). Prior to testing, the specimens were clamped with a clamping length of 20 mm, and to compensate for the clamping force that was applied, the specimens were elongated until the force was equal to zero. In the following, the initial length l_0_ of the specimen was measured and the displacement-controlled testing was performed under isothermal condition.

**Figure 2:**
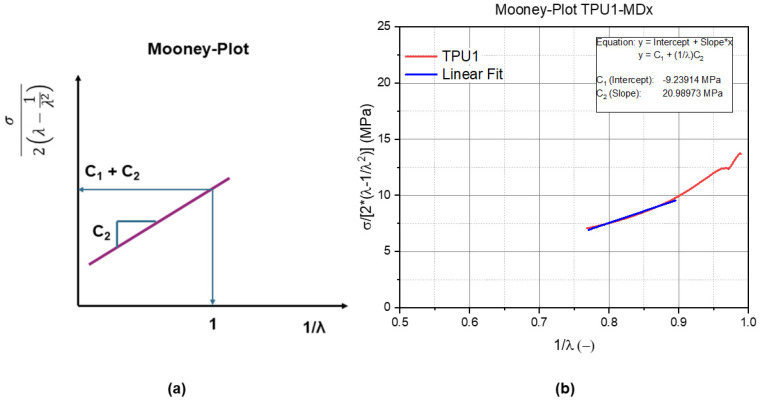
(**a**) Schematic representation of the Mooney plot; (**b**) Mooney plot and estimated material parameters for the constitutive law. The * illustrates a multiplication.

The material parameter determination was performed by assuming incompressibility and by measuring the uniaxial characteristics. As shown in Figure 2b, all of the experimental data were fitted iteratively to identify parameters *C*_1_ and *C*_2_ of the Mooney–Rivlin model (see Equation (1)) for *λ*_1_ = *λ* (stretch in loading direction); *λ*_2_ = *λ*_3_ = 1/(*λ*)^1/2^ is shown in Figure 2a.

(1) $$\frac{\sigma }{2\left(\lambda -\frac{1}{\lambda^{2}}\right)}=C_{1}+\frac{1}{\lambda }C_{2}$$

This model does not capture the temperature dependency of hyperelastic materials. Therefore, a dynamic thermomechanical analysis (DTMA) was performed to determine the temperature- and frequency-dependent E′ and E″ for the loss factor tanδ = E″/E′. The moduli were derived under the assumption of incompressibility according to DIN 53513:1990-03 by multiplying the measured specimen stiffness (dynamic force/dynamic displacement) with the shape factor (L_m_^2^/(L_0_ A_0_) with mean length L_m_ under static loading, initial length L_0_, and initial cross-section A_0_). The specimen geometry was a rectangular and had a width of 10 mm, a length of 35 mm, and a thickness of 3 mm. The dynamic thermomechanical behavior of the TPUs were analyzed under uniaxial loading at a temperature range from −80 °C to +80 °C and at frequencies from 0.5 Hz to 50 Hz. For the PDMS blends, the temperature range was from −50 °C to 50 °C at the same frequency range (0.5 Hz to 50 Hz). DTMA was performed with an Eplexor 500 N (Netzsch-Gerätebau GmbH, Selb, Germany) and started at the lowest temperature and was increased in steps of 5 K or 10 K, depending on the temperature-dependent modulus transition of the material.

**Figure 3:**
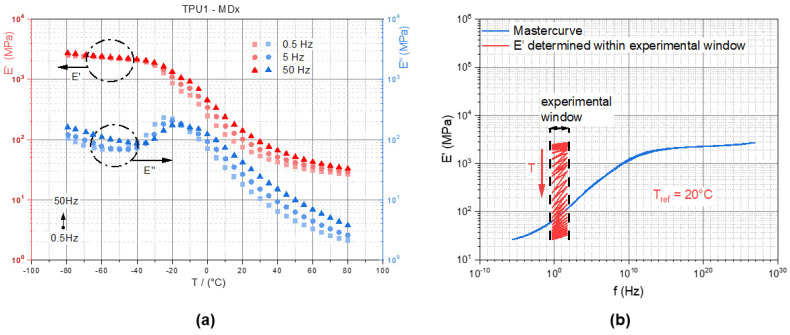
DTMA analyses for TPU1-MDx; (**a**) loading frequency and temperature-dependent storage and loss moduli (E′ and E″); (**b**) calculated master curve with a reference temperature of 20 °C.

The dynamic wave amplitude was a sine wave with mean strain levels of 20% (TPUs) and 8% (PDMS-blends) and dynamic peak-to-peak (p-p) amplitudes of 2% (TPUs) and 0.5% (PDMS-blends). Both parameter-sets were selected to characterize the materials within the linear viscoelastic regime. In order to compensate the thermal elongation, a holding force of 0.5 N was set, and the initial length at isothermal conditions was measured. At each testing temperature, pre-cycles under the same conditions as those mentioned above were performed for 1 s to avoid stress softening effects. Figure 3a shows the temperature dependent E′ and E″ at three excitation frequencies (0.5 Hz, 5 Hz, 50 Hz), and Figure 3b illustrates the frequency-dependent E′ master curve for the reference temperature T_ref_ of 20 °C. Additionally, the data from the measured experimental window are shown in Figure 3b, which reveal that E′ decreases as the temperature increases. According to the time–temperature superposition principle, the experimental data at lower temperatures are equivalent to high frequency data (shift to the right) and vice versa (cf. [19]).

**Figure 4:**
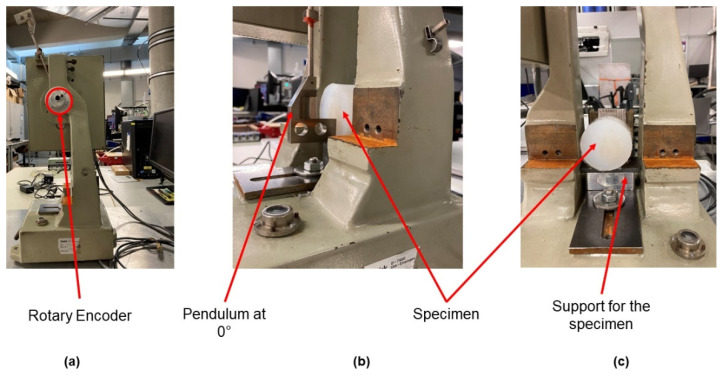
(**a**) Pendulum at start position; (**b**) pendulum and a PDMS blend at 0° position; (**c**) specimen and the clamping.

For the characterization of the damping capacity, the rebound resilience was measured. The rebound resilience (ratio of rebound height and initial drop height, *h*/*h*_0_) is related to the damping capacity (ratio of dissipated energy and elastic energy, U_d_/U_e_) and, in general, the higher the rebound resilience, the lower the damping of a material that is within the range of 0 (ideal damper) and 1 (ideal elastic). The measurement of the rebound resilience was performed with a custom-made test set-up that was based on a pendulum impact tester (ZwickRoell, Ulm, Germany) with a 0.5 J pendulum. The position of the pendulum was recorded by a rotary encoder (Kübler, Villingen-Schweninngen, Germany) connected to a GEN2i (Genisis High Speed Mainframe with integrated PC, HBM, Vienna, Austria) for data acquisition. The measurement procedure involved the pendulum being placed at a 0° position, and the specimen was placed at the center point of the pendulum at impact. Depending on the specimen size, the clamping base varied in both height and depth. The specimen was supported on the edgewise frontal surface and on the backside (see Figure 4). It was important that the specimen was placed and positioned so that the pendulum made contact with the specimen at the 0° position in order to maintain a higher accuracy impact speed. Finally, the measurement was performed by dropping the pendulum on the specimen, and this was repeated five times for each temperature.

The TPU specimens were rectangular plates (50 mm × 40 mm) with a thickness of 6.30 mm that had been injection moulded by DMH Dichtungs- und Maschinenhandel (Styria, Austria). The PDMS blends were casted cylinders with a diameter of ⌀60 mm and a height of 30 mm. The measurements were performed at −20 °C, 22 °C, and 60 °C for the TPUs and at −50 °C, −30 °C, −25 °C, 0 °C, 30 °C, and 50 °C for the PDMS blends. Different temperature ranges were selected for the examined materials, as TPU has a glass transition temperature Tg of −20 °C (see Figure 3a), limiting the low temperature applicability. The specimens were conditioned for min. 1 h at each temperature in the temperature chamber (CTS Clima Temperatur Systeme GmbH, Jennersdorf, Austria). Once the measurement was conducted, the angular position was recorded, and the height, impact speed, and impact force were derived. The conversion from the recorded angular position to the height of the pendulum is given in Equation (2). Subsequently, the rebound resilience was determined by the ratio of the rebound height h and initial height h_0_ of the pendulum (see Equation (3)). The rebound resilience is directly linked to the loss factor tanδ, and the relationship is given in [8]. For small tanδ values, the relationship is reduced to ln(*h*/*h*_0_) ≅ (1 − π tanδ).

(2) $$h=L-L*cos\left(B*\frac{\pi }{180}\right)$$

- *h*—actual height (m).
- *h*_0_—height of the start position (m).
- *L*—length of the pendulum (m).
- *B*—measured angular data (rad).

(3) $$rebound\;resilience=\frac{h}{h_{0}}$$

### 2.3. FE-Calculations

**Figure 5:**
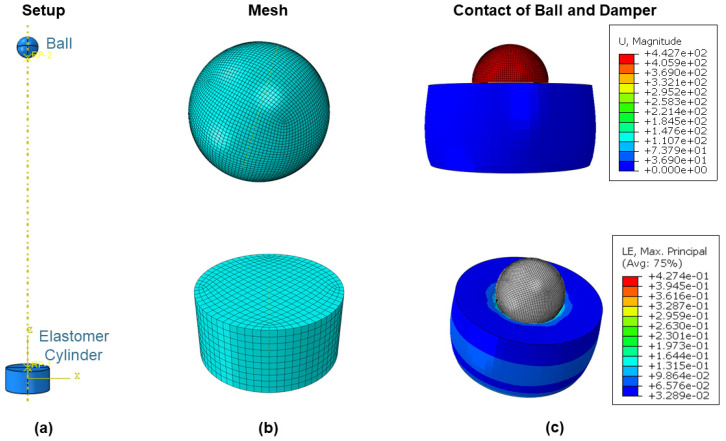
(**a**) Setup of the ball-drop test; (**b**) illustration of the meshed parts; (**c**) different viewports of the damper loaded with the ball.

The finite element (FE) simulation of a ball drop test configuration was performed using a ball with a diameter of 29 mm that was dropped onto a hyperelastic cylinder. The FE model was validated by the rebound resilience experiments. The model was set-up in Abaqus 2020 (Dassault Systèmes Simulia, Vélizy-Villacaublay, France) and is shown in Figure 5a. The initial position of the ball was 430 mm above the damper. With this height and the mass of the ball, the impact energy was equal to the experimental setup of the rebound resilience. The step was primarily set as dynamic and explicit, with a time of 1.5 s. For the ball, the interaction type was general contact (explicit). All of the part interactions were set to “all with itself”, with frictionless tangential behavior. The boundary conditions at the bottom part of the damper were fixed in all of the directions of freedom, and gravitational force (g = 9.81 m/s^2^) was applied in the z-direction.

Damping was modelled with Rayleigh damping (α, β parameters) [20], and hyperelastic material behavior was modelled with the Mooney–Rivlin constitutive law. Due to the large deformation of the rubber, the nonlinear geometric function was enabled. The ball was modelled as being of a linear elastic configuration and had the same parameters as steel (E = 210 GPa, ν = 0.33, ρ = 7830 kg/m^3^). For the ball as well as for the damper, an 8-node linear brick with reduced integration and hourglass control(C3D8R) was used (as shown in Figure 5b). The approximate global size was set to 3 mm for the damper and to 0.71 mm for the ball. Curvature control was used to apply the global seeds. The maximum deviation factor was set to 0. The default setting in Abaqus CAE is 1. Figure 5c shows the first impact of the ball on the damper. To validate the results, the displacement of the ball in the z-direction was used to calculate the rebound resilience according to Equation (3).

In the following, the results of the experimental and numerical analyses are presented, starting with the hyper- as well as viscoelastic (DTMA) characterizations followed by the material parameter determination for the Mooney–Rivlin constitutive model and the rebound resilience results. The rebound resilience results are compared to the simulated data as well as to the DTMA results.

## 3. Results

**Figure 6:**
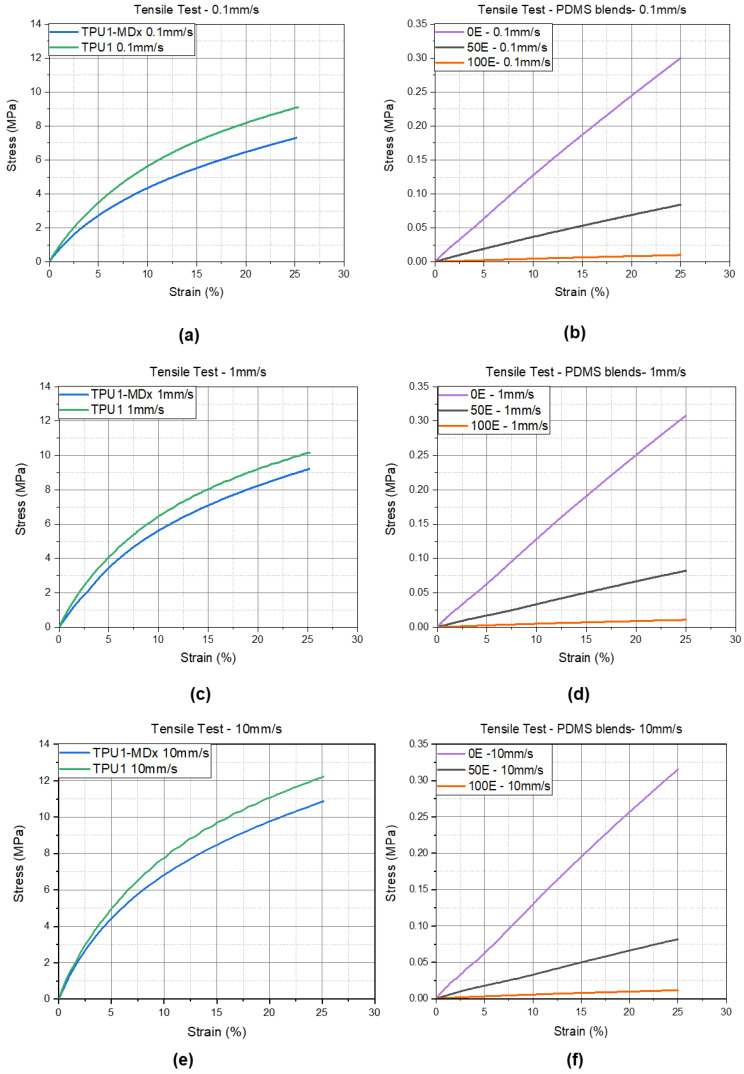
Stress–strain data from the evaluated tensile test of the TPU and PDMS mixtures. (**a**,**b**) 0.1 mm/s loading rate; (**c**,**d**) 1 mm/s loading rate; (**e**,**f**) 10 mm/s loading rate.

The stress–strain curves were necessary for the calculation of the Mooney–Rivlin parameters *C*_1_ and *C*_2_. For the calculation of *C*_1_ and *C*_2_, the data were reduced to 25% strain due to the fact that the accuracy of the Mooney–Rivlin constitutive law is higher at small strains. The diagrams in Figure 6a,c,e show the stress–strain curves of the examined TPUs, which behave very similarly. These results show that the iron particles in the TPU1-MDx have no influence on the tensile behavior.

In Figure 6b,d,f the stress–strain curves of the PDMS formulations are shown. For all of the loading rates, the 0E (100% Sylgard) was the stiffest material within the PDMS formulations. In general, the TPUs (see Figure 6) were stiffer than the PDMS (stress, σ of 0E at strain, ε = 25% (0.1 mm/s) = 0.30 MPa, σ of TPU1 at ε = 25% (0.1 mm/s) = 9 MPa). The stress–strain characteristic of the 50E blend is closer to that of the 100E but stiffer.

**Figure 7:**
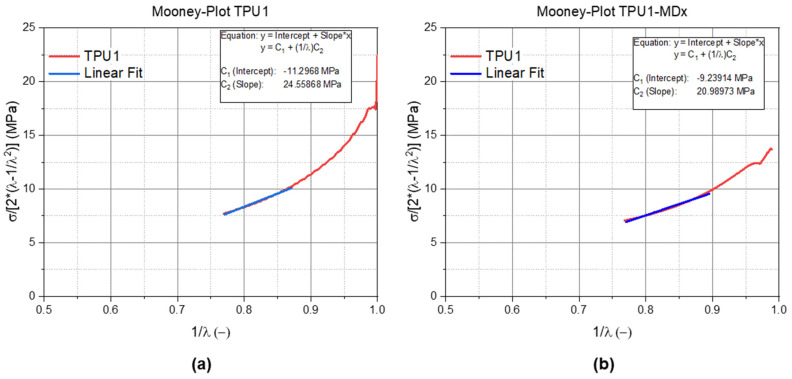
Mooney plot of the (**a**) TPU1 and (**b**) TPU1-MDx. The red curve illustrates the measured data, while the blue curve indicates the linear fit. The * illustrates a multiplication.

**Figure 8:**
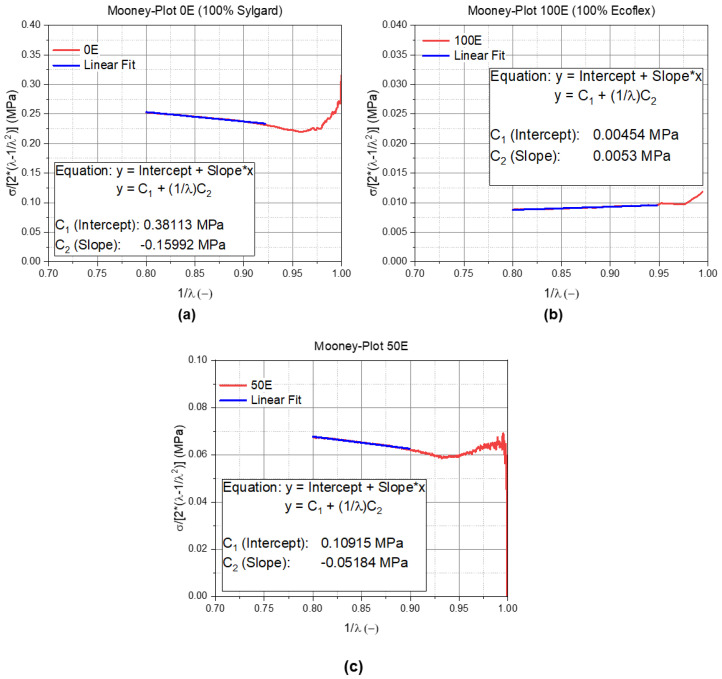
Mooney plot of the (**a**) 0E, (**b**) 100E, and (**c**) 50E blends. The red curve illustrates the measured data, while the blue curve indicates the linear fit. The * illustrates a multiplication.

The determined and validated Mooney–Rivlin material parameters are listed in Table 1 for the TPUs and in Table 2 for the PDMS formulations. These parameters were determined as explained earlier with the Mooney plot (Figure 2). The validation of the Mooney–Rivlin parameters are shown in Figure 7a,b for the TPU1 and for the TPU1-MDx blends and in Figure 8a for the 0E (100% Sylgard), in Figure 8b for the 100E (100% Ecoflex), and in Figure 8c for the 50E blends. In all of the diagrams, the red curve illustrates the measured data, and the blue one illustrates a linear fit, which describes the Mooney–Rivlin parameter.

**Figure 9:**
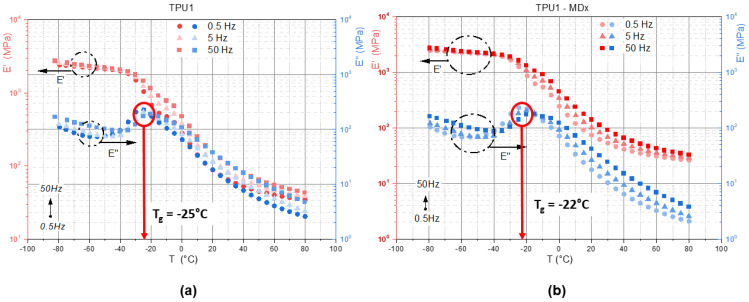
Storage and loss modulus (E′ and E″) over temperature. (**a**) TPU1; (**b**) TPU1-MDx.

The characterization of the loading rate and temperature dependency of the TPUs under dynamic thermomechanical loading confirmed that the Fe particles did not influence the behavior of the material significantly. In Figure 9a,b, the data for the storage (E′) and Young’s loss modulus (E″) of TPU1 and TPU1-MDx are shown. Similar characteristics were measured. Only the temperatures at the maxima of E″ were shifted, indicating that the glass transition temperatures were affected by the Fe particles. The TPU1 revealed the highest E″ at −25 °C (Figure 9a), while the maximum E″ of TPU1-MDx was at −22 °C (Figure 9b). As such, the glass transition of TPU1-MDx occurred at a higher temperature, thus limiting the low temperature resilience of the material.

**Figure 10:**
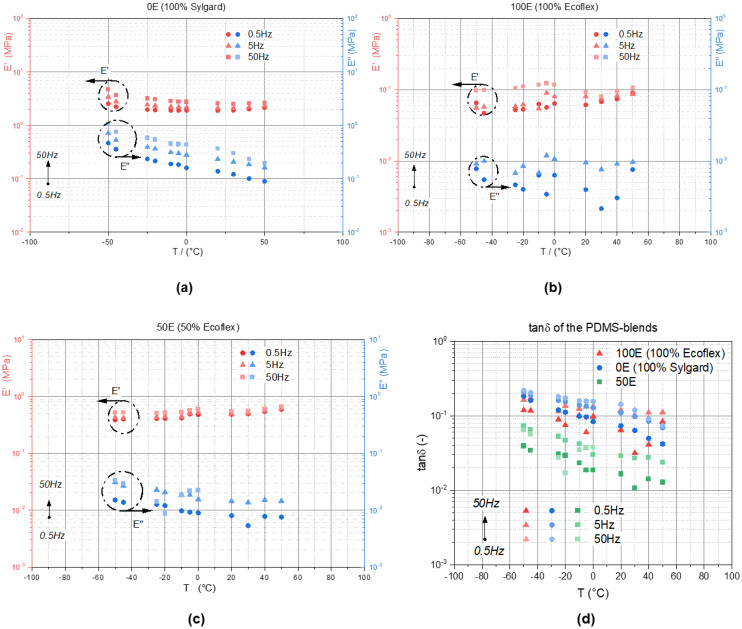
Storage and loss modulus (E′ and E″) over temperature: (**a**) 0E (100% Sylgard); (**b**) 100E (100% Ecoflex); (**c**) 50E. (**d**) illustrates the tanδ of all three PDMS-blends.

Figure 10a–c illustrate the storage (E′) and loss modulus (E″) over temperature for the 0E (**a**) the 100E (**b**) and the 50E (**c**) blends, respectively. Compared to the TPUs all of the PDMS-blends show a nearly constant E′ over the temperature. The 0E has the highest E′ followed by the 50E and the 100E blend. The loss modulus E″ of the 0E blend decreased with increasing temperature, while the 50E and the 100E blend revealed nearly a constant E″ across the experimental temperature range. While the maxima of E″ were obtained for all of the excitation frequencies at −50 °C for the 0E and the 50E blend, the 100E blend shows a maximum of E″ at 0 °C. At temperatures above 40 °C, the E″ of 100E for all of the frequencies approached the value of 0.01 MPa. This is an important insight that can be used for damper material selection in high-frequency vibration applications.

Figure 10d illustrates the loss factor tanδ (E″/E′) over the temperature for all three PDMS blends. Both neat PDMS (0E and 100E) show similar tanδ, which means that both have a similar damping capacity. The 50E blend shows a loss factor that is approximately a decade lower. This means that the damping capacity of the 50E blends are reduced compared to the neat silicone elastomers Sylgard and Ecoflex.

**Figure 11:**
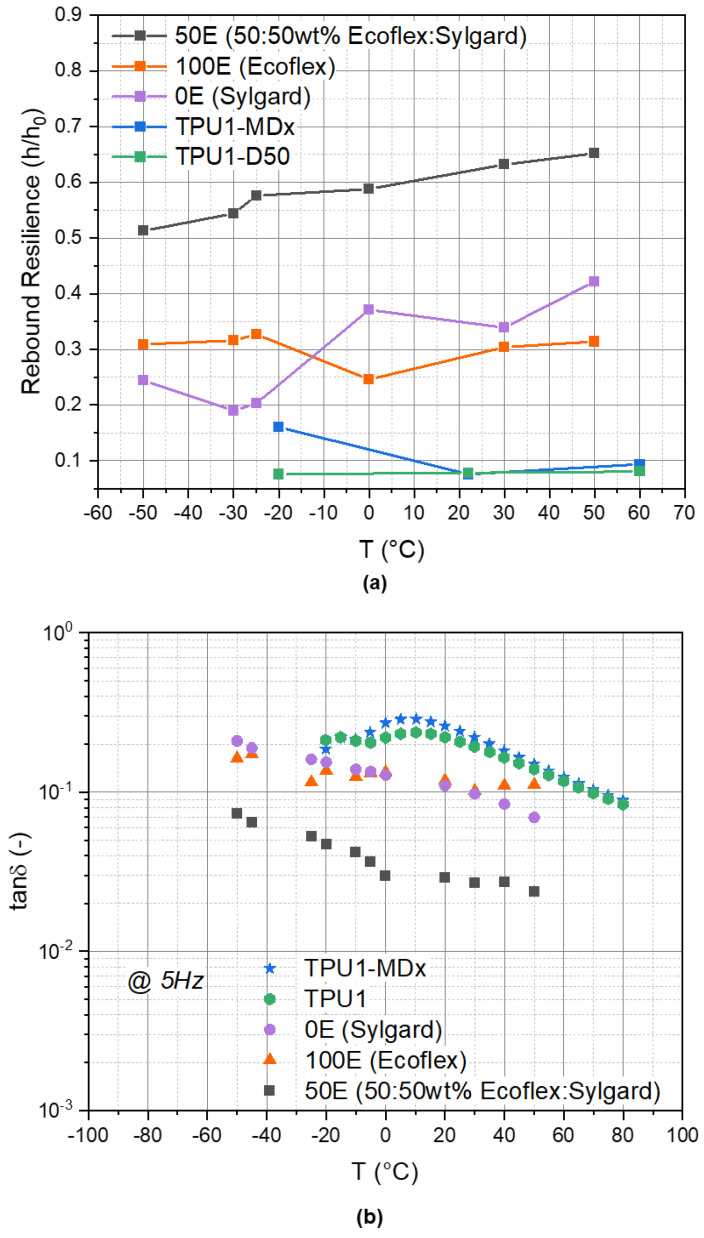
(**a**) Rebound resilience (h/h_0_) and (**b**) tanδ (E″/E′) of all characterized materials.

The results of the experimentally determined rebound resilience are shown in Figure 11a. In this diagram, the rebound resilience of the first rebound for each temperature is shown. In comparison, Figure 11b shows the predicted tanδ of all of the materials at 5 Hz, which is indirectly proportional to the rebound resilience. A high rebound resilience means a low tanδ and vice versa. The 50E PDMS blend has the highest rebound resilience of all of the different material formulations over the entire tested temperature range. This fits the result in Figure 11b, with the lowest tanδ for the 50E blend. Moreover, the DTMA of the 100E fits the results of the rebound resilience the best. Maximum E″ was achieved at 0 °C, so the rebound resilience is at a minimum at this temperature.

It is also interesting that the 0E blend has the lowest value at −30 °C, while the results of the DTMA revealed that the highest E″ is at −50 °C (see Figure 10a). This has to be analyzed in detail.

Generally, the rebound resilience of the 0E and 100E neat PDMS blends were much lower (i.e., higher damping capacity U_d_/U_e_) compared to that of the 50E blend. Furthermore, similar rebound resilience data were measured for the neat PDMS. This trend confirmed that the loss factor tanδ of both materials are similar within the range of 0.05 and 0.3, depending on the testing temperature (see Figure 11b). The Young’s moduli of the 100E (at 20 °C) and of 0E blends were in the range of 0.1 MPa and 1 MPa, respectively.

The TPUs exhibited similar rebound resilience behavior between 22 °C and 60 °C; however below −20 °C, their characteristics diverge significantly (see Figure 11a). This was in accordance with the shift of the glass transition temperature with the incorporation of Fe particles in TPU1 (cf. Figure 9a,b) and indicated the limitation of the low-temperature resilience of TPU1-MDx.

Overall, the TPUs revealed the best damping capacity (lowest rebound resilience or highest tanδ, respectively) of the characterized materials that were studied. For low-temperature applications up to −20 °C, TPU1 is a potential candidate material for dampers.

**Figure 12:**
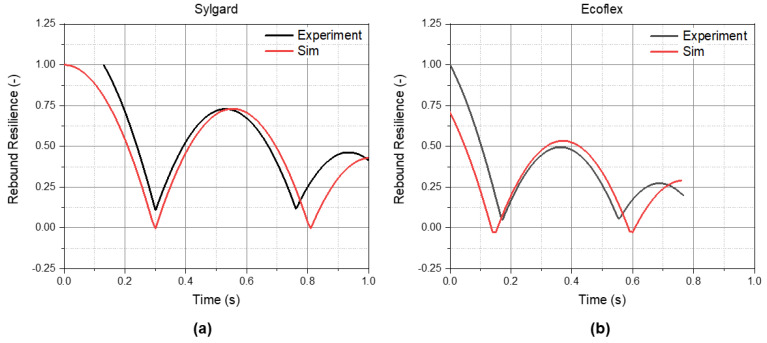
Comparison of the measured rebound resilience and the FE-calculated rebound resilience (**a**) for the 0E PDMS Sylgard and (**b**) for the 100E PDMS Ecoflex.

Using the material parameters that were determined experimentally, a FE simulation of the ball drop test configuration for the PDMS blends was conducted. In Figure 12a,b, the results of the 0E and 100E blends, respectively, are presented for comparative analyses. The black curve represents the measured rebound height, and the red curve represents the results of the simulation. Generally, good agreements were found between the FE and the experimentally determined data. For the 0E PDMS, the maximum rebound resilience of the experiment was 0.728, and the predicted resilience was 0.730. Only the impact time was shifted to the right by a small fraction of a second, about 0.023 s. For 100E, the experimental maximum rebound resilience was at 0.495, and the FE result was set at 0.535. However, the difference between the impact times from the simulation and from experiment was smaller compared to the simulation of the 0E blend (0.005 s).

## 4. Discussion

The results of the hyper- and viscoelastic characterizations for the TPUs showed that the Fe particles have a low amount of influence on the mechanical behavior of the TPU1-MDx. Additionally, the results of the experimentally determined rebound resilience were within the same range, except at temperatures below 20 °C. At this temperature, the TPU1 damps better than the TPU1-MDx. This was related to the shifted glass transition temperature of TPU1-MDx to higher temperatures with the incorporation of Fe particles. However, above 20 °C, the TPU1’s dynamic mechanical behavior remained unaffected by the Fe particles. Within a magnetic field, the Fe particles were magnetically polarized, reoriented within the softer matrix TPU and, ultimately, the macroscopic stiffness increased (magneto-rheological effect). Hence, this formulation is perfectly suitable as an active damping material but has to be experimentally validated in the future.

In [14], a formulation concept to increase the damping capacity of TPUs was presented. TPUs were filled with phenolic resin (PR) to increase the amount of hydrogen bonds (H-bonds). The unfilled TPU revealed comparable properties to those of the TPUs that were presented in this work; thus, this modification enables the damping behavior to be further improved and adjusted. This tailoring of the damping properties has to be investigated for Fe particle-reinforced TPU formulations in the future.

The uniaxial tensile tests of the PDMS blends revealed that the desired (tailored) blend properties can be achieved. The 0E (Sylgard) was the stiffest blend, and the 100E (Ecoflex) blend was the softest. Interestingly, the 50E blend (mixing ratio of 50:50) exhibited a tensile behavior that was more similar to that of the 100E blend rather than the 0E neat PDMS; however, it also demonstrated the highest rebound resilience values, indicating lowest damping capacity (U_d_/U_e_). This is a particularly important insight for tailoring material properties with low stiffness and low damping capacity requirements.

The presented results of this work are beneficial as initial optimization values for inverse methods. The presented moduli (E) of the Ecoflex in this work fits the E-values that were used as indicators for the applied inverse method in [15].

Furthermore, a FEA model was set based on the material parameters that were determined from the hyperelastic characterizations. The model was validated by the simulation of a (rigid) ball being dropped onto an hyperelastic cylinder. Good agreement was found between the simulation and the performed rebound resilience experiments. This model can further be utilized to dimension (engineer) as well as optimize damping structures.

The presented methods and results are also of particular interest tin terms of the insights gained into lattice structures made of (thermoplastic) elastomers with inherent viscoelasticity. Dong et al. [1] investigated different TPU lattice structures developed for shoe soles through numerical and experimental compressive loading. In order to exploit the TPU to its full potential, the structures had to be characterized with the presented methodology presented in that paper in order to optimize the lattice structure of shoe soles for customer needs (e.g., for certain temperatures or frequencies).

## 5. Conclusions

The potential of smart materials to reduce the complexity of state-of-the-art products by exploiting their function integration capability is a vivid field of research. New generations of products benefit from smart materials and product engineering have to be adapted in order to fully utilize the functions that these materials provide. This study only considered passive damping; however, the possibility of exposing the Fe particle-filled material to magnetic fields must be analyzed in a case study under realistic loadings. Interestingly, the findings of the applied methodology revealed that the material behavior of TPU is not compromised by compounded Fe-particles. Strength and failure analyses must be conducted in the future to determine the structural integrity of dampers that are made using this material formulation. However, the presented systematic methodology, including the data reduction, as well as the material parameter determination methods provided temperature and excitation frequency-dependent material parameters of the characteristics for product engineering and for the optimization of dampers with FE analyses.

## Figures and Tables

**Table 1:** Determined material parameters of the TPUs.

Mooney–Rivlin Parameter	TPU1	TPU1-MDx
*C*_1_	−11.30	−9.24
*C*_2_	24.56	20.99

**Table 2:** Determined material parameters of the PDMS formulations.

Mooney–Rivlin Parameter	0E (100% Sylgard)	50E (50 wt% Ecoflex)	100E (100% Ecoflex)
*C*_1_	0.38	0.11	0.0045
*C*_2_	−0.16	−0.05	0.0053

## Data Availability

Not applicable.
